# Molecular Changes in the Non-Inflamed Terminal Ileum of Patients with Ulcerative Colitis

**DOI:** 10.3390/cells9081793

**Published:** 2020-07-28

**Authors:** Ho-Su Lee, Maaike Vancamelbeke, Sare Verstockt, Tom Wilms, Bram Verstockt, João Sabino, Marc Ferrante, Séverine Vermeire, Isabelle Cleynen

**Affiliations:** 1 Laboratory of Complex Genetics, Department of Human Genetics, KU Leuven, 3000 Leuven, Belgium; hosu.lee@student.kuleuven.be (H.-S.L.); sare.verstockt@kuleuven.be (S.V.); tom.wilms@kuleuven.be (T.W.); 2 Department of Biochemistry and Molecular Biology, University of Ulsan College of Medicine, Seoul 05505, Korea; 3 Translational Research Center for Gastrointestinal Disorders (TARGID), Department Chronic Diseases, Metabolism & Ageing (CHROMETA), KU Leuven, 3000 Leuven, Belgium; maaike.vancamelbeke@hotmail.com (M.V.); bram.verstockt@kuleuven.be (B.V.); joao.sabino@uzleuven.be (J.S.); marc.ferrante@uzleuven.be (M.F.); severine.vermeire@uzleuven.be (S.V.); 4 Department of Gastroenterology and Hepatology, University Hospitals Leuven, KU Leuven, 3000 Leuven, Belgium

**Keywords:** mucosal gene expression, RNA-Seq, small bowel dysfunction, ulcerative colitis

## Abstract

Ulcerative colitis is a chronic inflammatory disease confined to the colon. Although the etiopathogenesis remains unknown, small bowel dysfunctions like histological and permeability alterations have been described in ulcerative colitis. We evaluated the molecular gene signature in the non-inflamed terminal ileum of 36 ulcerative colitis patients (7 active, with Mayo endoscopic subscore ≥2, and 29 inactive) as compared to 15 non-inflammatory bowel disease controls. Differential gene expression analysis with DESeq2 showed distinct expression patterns depending on disease activity and maximal disease extent. We found 84 dysregulated genes in patients with active extensive colitis and 20 in inactive extensive colitis, compared to controls. There was an overlap of 5 genes: *REG1B*, *REG1A*, *MUC4*, *GRAMD2*, and *CASP10*. In patients with left-sided colitis, ileal gene expression levels were similar to controls. Based on gene co-expression analysis, ileal changes in active ulcerative colitis patients were related to immune functions. The ileal changes in the inactive ulcerative colitis subjects converged into the maintenance of the intestinal barrier through increased mitochondrial function and dampened immune functions. In conclusion, we identified molecular changes in the non-inflamed ileum of ulcerative colitis that are dependent on colonic inflammation.

## 1. Introduction

Inflammatory bowel diseases (IBD), comprising ulcerative colitis (UC) [1] and Crohn’s disease (CD), are chronic disorders of the gastrointestinal tract, characterized by relapsing gut inflammation [2]. UC is limited to the colon and is characterized by a continuous mucosal inflammation, whereas CD can affect all parts of the gastrointestinal tract and is determined by a non-continuous transmural inflammation [2]. From a pathophysiological point of view, there is currently no explanation for the spatial restriction of the inflammation in UC [3]. Small bowel dysfunction has, however, been reported in UC [3]. For example, histological alterations including structural alterations of villi and inflammatory cell infiltration [1,4,5,6,7], and increased intestinal permeability, have been detected in the small bowel of patients with UC [8,9]. There is also experimental evidence of small bowel dysfunction from animal models of colitis. During experimental colitis, alterations in small bowel permeability and brush border enzymatic activity are observed [7,10,11]. Moreover, in IL10-deficient mice which spontaneously develop colitis, colitis is mitigated by reducing small intestinal permeability upon treatment with the zonulin peptide inhibitor AT-1001 [12]. This again suggests a possible link between small bowel dysfunction and colonic inflammation. To date, however, the precise mechanisms underlying this dysfunction have not been elucidated. 

The intestinal transcriptomes of IBD patients have been previously analysed to gain insights into the molecular mechanisms leading to disease development [13,14]. Likewise, genome-wide expression profiling of unaffected terminal ileum tissue from patients with UC can provide better insights into the molecular characteristics of small bowel dysfunction in these patients. To the best of our knowledge, only two studies report transcriptomic data of unaffected terminal ileum in UC, albeit on at least partially overlapping datasets, and with the primary goals not directed towards finding the molecular alterations in UC terminal ileum [15,16]. They found significant alterations in gene expression levels in the terminal ileum of UC patients, with Dual Oxidase Maturation Factor 2 (*DUOXA2*) and Dual Oxidase 2 (*DUOX2*) being among the most significant [15,16]. The *DUOX2/DUOXA2* complex plays an important role in maintaining mucosal immune homeostasis [17,18,19] and in the host-microbial interaction in IBD [16]. They did not investigate whether these alterations in the non-inflamed small bowel are related to the disease activity and/or disease extent of UC.

We performed RNA sequencing on mucosal non-inflamed terminal ileum tissue samples from active and inactive UC patients, as well as non-IBD controls, to identify if and which genes and gene co-expression modules were dysregulated. The two primary aims of our study were (i) to investigate the molecular alterations in non-inflamed ileum tissue in UC, and (ii) to determine whether these alterations are dependent on colonic inflammation. The latter was assessed in terms of disease activity and disease extent. 

## 2. Materials and Methods

### 2.1. Study Subjects and Samples

Patients with an established diagnosis of UC [20] were included. Terminal ileum mucosal biopsies were obtained during endoscopy from macroscopically unaffected sites in 37 UC patients. Colonic inflammation activity at endoscopy was determined in accordance with the Mayo endoscopic subscore, with active colitis defined by a value of 2 or 3 [21]. The maximal UC disease extent during follow-up and at the time of endoscopy was categorized in accordance with the Montreal classification and based on macroscopic findings [22]. Sixteen non-IBD controls were included for comparison. These underwent endoscopy for polyp screening, and had normal mucosa without evidence of gut inflammation during endoscopy. The ethics committee of the University Hospitals Leuven approved the study on 29 March 2012 (Approval number: B322201213950/S53684). All patients provided written informed consent.

### 2.2. RNA Isolation and Sequencing

Ileal biopsies were immediately placed in RNAlater (Ambion, Austin, TX, USA). The total RNA was extracted using the AllPrep DNA/RNA Mini kit (Qiagen, Hilden, Germany) in accordance with the manufacturer’s instructions. RNA integrity and quantity were assessed using a 2100 Bioanalyzer (Agilent, Waldbronn, Germany) and a Nanodrop ND-1000 spectrophotometer (Thermo Scientific, Waltham, MA, USA). Extracted RNA was stored at −80 °C until further processing. Single-end RNA sequencing was conducted using an Illumina HiSeq 4000 system (Illumina, San Diego, CA, USA) in accordance with the manufacturer’s instructions. Raw RNA sequencing data were aligned to the reference genome using Hisat2 version 2.1.0 [23], and absolute counts were generated using HTSeq [24]. Non-coding transcripts or low-expressed transcripts with an average count ≤10 were excluded from further analysis, leaving 14,025 transcripts. Principal component analysis (PCA) was used to identify outliers and to check for clustering of samples. We excluded two outlier samples (one UC patient and one control) based on PCA (Supplementary Figure S1A), leaving 36 UC patients (61% male; median age 52 years) and 15 non-IBD controls (40% male; median age 55 years) for further analysis (Table 1 and Supplementary Figure S1B). PCA was performed using the FactoMineR package [25]. Grouping phenotypes (case versus non-IBD control, with and without cases broken down in disease activity and/or disease extent) were added as supplementary variables in the analysis, in order to calculate their correlation with the obtained expression-based PCs. 

### 2.3. Differential Gene Expression Analysis 

Absolute counts were analysed for differential gene expression between cases and controls using the DESeq2 (version 1.20.0) package [26] in R (version 3.5.1), and adjusted for age and sex. Differentially expressed genes were defined by an absolute log_2_ fold-change ≥1 (i.e., fold-change ≥2) and a corrected *p*-value (Benjamini–Hochberg method [27]) below 0.05. We reported corrected *p*-values for differential gene expression analysis unless noted otherwise.

### 2.4. Quantitative Real-Time Polymerase Chain Reaction (qRT-PCR) 

To validate RNA-seq data, gene expression for selected differentially expressed genes in ileal mucosal biopsies was studied trough a qRT-PCR analysis. One sample of active extensive UC was unavailable for qRT-PCR. cDNA was synthesised from 0.5 μg RNA using the RevertAid H Minus First Strand cDNA synthesis kit (Fermentas, St. Leon-Rot, Germany), following the manufacturer’s protocol. To facilitate the analysis of multiple targets, we preamplified the cDNA (1:10 dilution) with a multiplex cDNA preamplification using the TaqMan PreAmp Master Mix kit (Applied Biosystems, Foster City, CA), according to the manufacturer’s protocol. The selected genes included *DUOXA2*, *DUOX2*, *REG1B*, *REG1A*, *MUC4*, *GRAMD2*, and *CASP10*, with *ACTB* and *GAPDH* as endogenous reference genes. An overview of the validated genes and the corresponding TaqMan Assay ID are given in Supplementary Table S1 (Thermo Fisher Scientific, Massachusetts, MA). qPCR was performed in a final reaction volume of 10 µL on a QuantStudio^TM^ 12K Flex Real-Time PCR system (Thermo Fisher Scientific, Massachusetts, MA). All samples were analysed in triplicate. mRNA levels were normalized to the geometric mean of the two housekeeping genes (*ACTB* and *GAPDH*) and quantified using the comparative (ΔΔ) Ct method.

### 2.5. Weighted Gene Co-Expression Network Analysis 

To identify modules of co-expressed genes, we applied the Weighted Gene Correlation Network Analysis (WGCNA, version 1.66) [28] using R (version 3.5.1). We used the same design as applied for differential gene expression analysis for data input for WGCNA: briefly, we normalized the counts and corrected for age and sex using the DESeq2 package. We constructed co-expression modules by calculating a matrix of the Pearson correlations between all gene pairs. To identify modules of highly co-regulated genes, we used average linkage hierarchical clustering to group genes based on the topological overlap of their connectivity, followed by a dynamic cut-tree algorithm to dynamically separate clustering dendrogram branches into gene modules (using deepSplit = 2 and a minimal module size of 30). A soft-threshold power value of 4 was selected in this analysis, which corresponds to a 87.2% scale-free topology. Each module was assigned a unique colour identifier. 

To identify which modules are most relevant in the context of colonic inflammation, each module was tested for correlation with phenotypes of interest (disease activity, disease extent…). For this analysis, each module is represented by its eigengene, i.e., the first principal component of the expression patterns of all genes within a given module, summarized into a single characteristic. We calculated the corrected *p*-value using the Benjamini–Hochberg method [27].

### 2.6. Pathway Analysis 

Ingenuity Pathway Analysis (IPA, QIAGEN Inc., www.qiagen.com/ingenuity) and the Reactome pathway database (www.reactome.org, version 68) were used to identify upstream regulators and canonical pathways associated with the differentially expressed genes, and with genes within each WGCNA module. To further evaluate the biological relevance of the differentially expressed genes and the genes within the modules, we also performed a gene ontology (GO) enrichment analysis of biological processes using PANTHER tools version 15.0 (pantherdb.org/tools/) [29]. A corrected *p*-value < 0.05 was considered as significantly enriched.

### 2.7. Statistical Analysis

Continuous variables were expressed as median and interquartile range (IQR), and categorical variables as frequencies and percentages. Data were compared using the Mann–Whitney U test for continuous variables, and with Fisher’s exact or Chi^2^ test for categorical variables. A *p*-value < 0.05 was considered statistically significant. Statistical analyses were done using R (version 3.5.1).

## 3. Results

### 3.1. Baseline Characteristics 

The baseline characteristics of the included patients and controls are listed in Table 1. Of the 36 UC patients, 7 (19.4%) had active colonic disease at the time of endoscopy (5 with an endoscopic Mayo score of 2, and 2 with an endoscopic Mayo score of 3), and 29 (80.6%) were inactive. The maximal extent of disease throughout follow-up in the active UC cases was left-sided colitis in 2 patients (28.6%) and extensive colitis in 5 patients (71.4%). At the time of endoscopy, among the active UC cases, 4 patients had left-sided colitis, and 3 had extensive colitis. The maximal disease extent among the inactive UC cases included 4 cases of proctitis, 11 left-sided colitis, and 14 extensive colitis. At time of endoscopy, 1 active UC patient (14.3%) and 13 inactive UC patients (44.8%) received biologics.

### 3.2. Gene Dysregulation in the Normal Terminal Ileum of UC Patients is Dependent on Colonic Inflammation

Based on PCA, which characterized the general gene expression differences among the 51 samples, we did not see any obvious clusters in the data (Supplementary Figure S1B). The first two principal components explained 35.6% of variance between the samples (26.3% for PC1, 9.3% for PC2). The highest correlation with PC1 was seen for the combination of disease activity and extent (*r*^2^ = 0.14, *p* = 1.21 × 10^−1^), while disease extent (irrespective of disease activity) was significantly correlated with PC2 (*r*^2^ = 0.13, *p* = 3.18 × 10^−2^). This suggested disease activity and/or disease extent contribute to the gene expression pattern in the terminal ileum in UC, albeit a small one.

**Figure 1:**
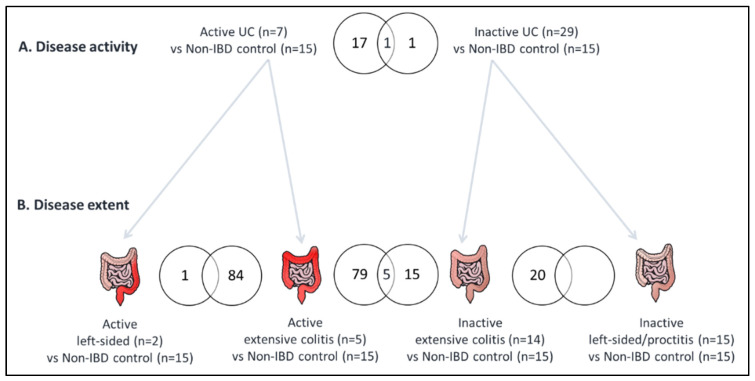
Overview of significantly dysregulated genes in the different comparisons. Venn diagrams of the differentially expressed genes in the comparative analysis of (**A**) active or inactive ulcerative colitis (UC) patients and non- inflammatory bowel disease (IBD) controls; and of (**B**) Extensive UC or left-sided colitis/proctitis and non-IBD controls, in active and inactive UC separately. The number of significantly differentially expressed genes in each comparative analysis is shown in circles. The number of overlapping genes between comparisons is also indicated.

We then separately compared gene expression in normal ileum of active and inactive UC patients with non-IBD controls (Figure 1, Table 2, Supplementary Figure S2A, and Supplementary Table S2). In active UC, we identified 18 differentially expressed genes, with *DUOXA2* being the most significant (log_2_ fold-change = 4.9, *p* = 5.58 × 10^−3^). The cytokines IL22, STAT1, and IFN-γ were the top 3 predicted upstream regulators of some of these 18 genes: IL22 for *MUC1* and *NOS2*; STAT1 for *DUOX2*, *MUC1*, and *NOS2*; and IFN-γ for *DUOX2*, *DUOXA2*, *MUC1*, and *NOS2* (Supplementary Table S3). In inactive UC, we identified two dysregulated genes compared to non-IBD controls: *REG1B* (log_2_ fold-change = 2.8, *p* = 3.42 × 10^−2^) and *CEBPD* (log_2_ fold-change = 1.1, *p* = 2.12 × 10^−2^). *REG1B* was also one of the 18 dysregulated genes found in active UC, but with more pronounced dysregulation in active than in inactive UC (active UC: log_2_ fold-change = 4.2, *p* = 1.98 × 10^−2^; inactive UC: log_2_ fold-change = 2.8, *p* = 3.42 × 10^−2^) (Table 2, Supplementary Figure S2A). 

**Figure 2:**
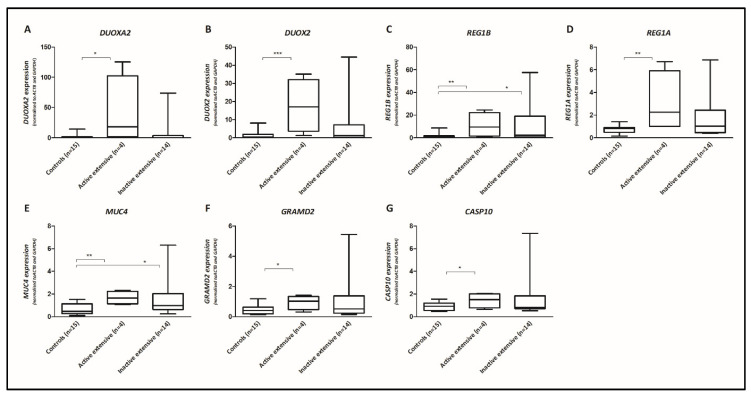
Validation of expression levels of seven selected mRNAs in ileal mucosal biopsies. Boxplots of the relative expression level of (**A**) *DUOXA2*, (**B**) *DUOX2*, (**C**) *REG1B*, (**D**) *REG1A*, (**E**) *MUC4*, (**F**) *GRAMD2*, and (**G**) *CASP10* in controls (*n* = 15), active extensive UC (*n* = 4), and inactive extensive UC (*n* = 14) patients, as assessed by qRT-PCR (box, 25–75%; whisker, min-max). The expression levels are normalized to the geometric mean of *ACTB* and *GAPDH*. Data were compared using the one-tailed Mann–Whitney U test (solid line with down angle, * *p* < 0.05; ** *p* < 0.01; *** *p* < 0.001).

We next studied if gene expression profiles in the terminal ileum depend on maximal disease extent. We therefore separately performed a differential gene expression analysis for extensive UC and left-sided colitis/proctitis, in active and inactive UC (Figure 1, Table 3, and Supplementary Table S2). We found 84 differentially expressed genes in active extensive UC compared to non-IBD controls, and 20 in inactive extensive UC, with an overlap of 5 genes (*REG1B*, *REG1A*, *MUC4*, *GRAMD2*, and *CASP10*; Figure 1, Table 3, Supplementary Figure S2B, and Supplementary Table S2). In line with the RNA sequencing results, qRT-PCR validation found significantly increased expression levels of each of these 5 genes in active extensive UC (Figure 2), as well as for *DUOXA2* and *DUOX2*, which were also among the top dysregulated genes for active UC (Table 2). In inactive extensive UC, increased *REG1B* and *MUC4* expression levels were validated using qRT-PCR (Figure 2). Although *REG1A*, *GRAMD2*, and *CASP10* genes could not be confirmed with qRT-PCR, we observed borderline significance for *REG1A* and *GRAMD2* (*p*-value = 0.09 and 0.13, respectively), and the observed *p*-values for all genes where concordant with their rank observed for RNA sequencing data (Table 3). 

IL1A, NFkB (complex), and PPRC1 were the top 3 predicted upstream regulators of 7, 8, and 4 out of 84 genes, respectively (Supplementary Table S3). The top 3 predicted upstream regulators in inactive extensive colitis were miR-155-5p, SOCS1, and TREM1, regulating *CXCL2* and *SOCS1*; *CXCL2* and *SOCS1*; and *CXCL2*, *HES4*, and *TIFA*, respectively (Supplementary Table S3). Given the number of dysregulated genes, we only performed pathway analyses on the 84 differentially expressed genes in active extensive UC. Among these 84 genes, 6 genes were involved in “Endothelin-1 Signalling” (*p* = 1.51 × 10^−4^), and 5 in “Granulocyte Adhesion and Diapedesis” (*p* = 5.50 × 10^−4^) (Supplementary Table S4). Significantly enriched biological processes among the dysregulated 84 genes were the regulation of inflammatory response, cell wall disruption in another organism, and antimicrobial humoral immune response mediated by antimicrobial peptide, hereby implicating the role of the antimicrobial response in the ileum of UC patients (Supplementary Table S4).

Only *LCT* (Lactase) was dysregulated in active UC with left-sided colitis (log_2_ fold-change = −5.3, *p* = 2.25 × 10^−2^, Supplementary Figure S2C). It should however be noted that these were only two patients. No significant differences were seen between inactive UC patients with left-sided colitis/proctitis (*n* = 15) and non-IBD controls (Supplementary Figure S2C). The analysis of extensive UC at the time of endoscopy in active UC patients showed similar findings both for extensive UC and maximal disease extent. 

### 3.3. Co-Expression Modules Are Associated with Colonic Inflammation

Besides looking at the differential gene expression of individual genes, we assessed whether we could find modules of genes with a similar ileal expression pattern. The WGCNA analysis found 39 co-expression modules (Supplementary Figure S3) that ranged in size from 60 to 1194 genes. None of these modules were significantly correlated with the studied traits after correction for multiple testing (corrected *p*-value < 0.05). Six modules showed a nominal significant (uncorrected *p*-value < 0.05) correlation with disease: three with inactive UC and three with active UC (Supplementary Figure S3). Of the three modules, one had a significant correlation with inactive UC, while two were positively correlated (colour code: purple and grey60) and included genes involved in mitochondrial translation and actin cytoskeleton, respectively; and one was negatively correlated (colour code: violet) and included genes involved in signal regulatory protein family interactions and lymphotoxin β receptor signalling (Supplementary Table S4 and Supplementary Table S5). These modules were found to correlate irrespective of maximal disease extent during follow-up (left-sided colitis/proctitis or extensive colitis). Of the 20 differentially expressed genes in inactive extensive UC, 6 were assigned to the purple module (Supplementary Table S5).

The three modules most correlated with active UC (colour code: saddlebrown, steelblue, and brown) were all positively correlated and mainly involved in immune functions (e.g., IFN and cytokine signalling, NFkB signalling, and antigen presentation) (Table 4, Supplementary Table S4). The most significantly enriched GO biological processes were the defence response to virus, protein deubiquitination, and regulation of biological quality, respectively. The frequently enriched GO pathways were the microbial response and immune reactions (Supplementary Table S4). Importantly, unlike in patients with inactive disease, this correlation seemed to be driven by maximal disease extent, as a significant correlation was only observed for extensive disease and not with left-sided disease extent during follow-up (Table 4, Supplementary Figure S3). The small sample size of these subgroups should however preclude from drawing firm conclusions. Similar results were obtained when we applied the disease extent at the time of endoscopy for patients with active disease (Supplementary Figure S3). Among 84 dysregulated genes in active extensive UC, 29 genes were assigned to the saddlebrown (*n* = 18) and brown (*n* = 11) modules (Table 4).

## 4. Discussion

**Figure 3:**
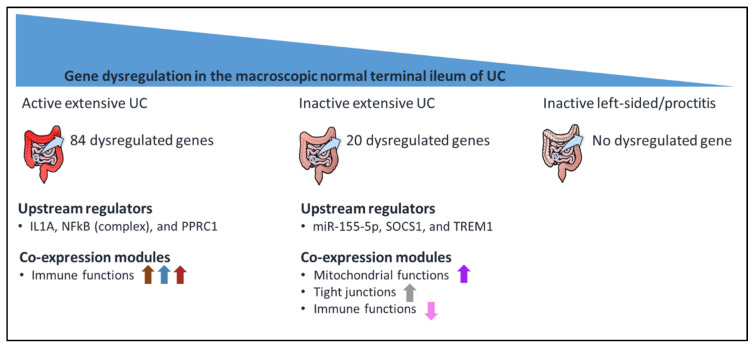
Schematic summary of the current study. Ileal expression from ulcerative colitis (UC) patients is dependent on UC disease activity and extent. The strongest dysregulations were seen in patients with extensive disease as maximal disease extent during follow-up. Co-expression module analysis found that ileal changes in active extensive UC are mainly related to the immune function (colour code for identified co-expression modules: saddlebrown, steelblue, and brown). Ileal changes in inactive UC, on the other hand, seem to be functioning to maintain the intestinal barrier with increased mitochondrial functions (colour code for identified co-expression modules: purple and grey60) and dampened immune functions (colour code for identified co-expression module: violet).

We found molecular alterations in the non-inflamed ileum of UC patients, which were dependent on the presence of colonic inflammation and disease extent, and thus point to a cross-talk between colon and ileum (see Figure 3 for an overview of the main findings). While patients with inactive colitis showed a similar gene expression profile in the ileum as non-IBD controls, more alterations in ileal gene expression were seen in patients with active colitis at the time of endoscopy. Patients who had previously experienced extensive disease showed a distinct gene expression pattern at the ileal mucosa, regardless of disease activity in the colon. 

When interpreting the results, we should keep in mind the potential limitations of our study. First, the current analysis is limited by the small sample size of our cohort, particularly for active left-sided disease, where we only have two samples. Further studies with a larger sample size are required to verify the present observations. Second, we acknowledge the possibility of confounders such as smoking or different medication influencing our results, although in our study we did not observe an influence of the use of biologics on PCA clusters. We also conducted a differential gene expression analysis including the use of biologics as a covariate and found that the obtained results were largely the same as the current results (data not shown). Third, it would have been interesting to have included the paired colonic mucosal tissue samples from the included patients, as these provide insight into the relationship between molecular alterations at the terminal ileum and colonic inflammation. These samples, however, were not available. Fourth, although the results of our study are supported by previous multiple lines of evidence that the identified genes do have biological relevance for IBD, we lack the functional analysis to specifically address the biological consequences of the identified genes. More mechanistic studies are needed to further demonstrate the precise mechanism by which the dysregulated genes influence the development of UC. There are also some strengths that we want to highlight. To the best of our knowledge, we are the first to specifically report on the molecular changes in the unaffected terminal ileum in UC patients and the relationship of these changes with the presence and extent of colonic inflammation, based on high-throughput mucosal transcriptional profiling. Besides, in addition to performing simple and well-established differential gene expression analyses, we used an unsupervised bioinformatics approach (WGCNA) to analyse gene co-expression networks.

We demonstrated that both the presence of colonic inflammation and the disease extent were associated with transcriptional alterations of the terminal ileum. Comparative analyses of gene expression levels in ileal biopsies of active UC patients versus non-IBD controls identified the significantly different expression of 18 genes. By contrast, patients with inactive colitis had a similar ileal gene expression signature to non-IBD controls. Importantly though, in both active and inactive colitis patients, the strongest dysregulations were seen in patients with extensive disease as maximal disease extent during follow-up. In the group of patients with active and extensive disease, 84 genes were significantly dysregulated in the terminal ileum, and with the strongest fold changes. Interestingly, the patients who previously had extensive colitis, though without colonic inflammation at the time of endoscopy, presented a distinct expression pattern from patients who previously had left-sided colitis. This suggests that the non-inflamed terminal ileum of UC patients does not restore to the non-IBD control expression pattern after the macroscopic resolution of extensive inflammation. Rather, these patients still show transcriptional alterations in their terminal ileum, although they are largely different from the alterations seen in active extensive disease patients, with an overlap of only 5 of the 20 genes. In particular, *CEBPD* showed a significant up-regulation in inactive extensive UC patients compared with controls. CEBPD is known to be induced in many inflammation-related diseases [30] and plays a possible anti-inflammatory role [31,32,33]. In a *Cebpd*-knockout mouse model, significant increases in oxidative stress and mitochondrial dysfunction were induced by radiation [34,35]. However, the role of these alterations in the context of chronic colonic inflammation in UC remains unclear. 

The most significantly differentially expressed gene in the terminal ileum of UC patients with active colitis was *DUOXA2*. This gene is known to be required for the maturation and function of *DUOX2*, which was also differentially expressed in active UC. This increased *DUOXA2* and *DUOX2* expression confirmed earlier findings where both genes were found to be increased in the ileum of both UC and CD [15]. The *DUOX2*/*DUOXA2* complex plays a role in maintaining mucosal immune homeostasis by the hydrogen peroxide-based epithelial defence [17,18,19] and in the interaction between host and microbiome [16]. In addition, the potential role of *DUOX2* in maintaining the epithelial barrier in the small bowel has been described in a *Duoxa*-knockout mouse model [36], and in patients with CD [37]. Another interesting pair of genes from our analyses is *REG1A/REG1B*. We observed a universal upregulation of *REG1A/REG1B* across subtypes of UC, including both active extensive colitis and inactive extensive colitis. REG1 proteins are involved in cell differentiation and proliferation within the gastrointestinal tract [38,39], and have been implicated in intestinal inflammation. For example, they showed upregulation during intestinal inflammation in infectious colitis [40]; were found to be up-regulated in metaplastic Paneth cells in both un-inflamed and inflamed colonic mucosa from IBD patients, with increased expression during inflammation [41,42]; and *REG1A/REG1B* was also found to be widely expressed in a newly identified T-cell subtype isolated from CD patients using a single-cell analysis of intestinal T cells [43]. Finally, genes *MUC1* and *MUC4* encode membrane-bound mucins from epithelial cells which aid in the intestinal mucosal barrier function [44,45,46]. Previously, our group found a possible critical role of epithelial barrier genes, including *MUC1* and *MUC4*, in IBD [47]. Particularly, *MUC1* and *MUC4* showed increased expression levels compared with controls not only in active CD and UC, but also in uninflamed CD ileum and IBD colon. Furthermore, previous genome-wide association studies [48] have identified the *MUC1* locus as a susceptibility locus for IBD, with additional evidence from expression quantitative trait loci data (GTEx Analysis Release V8, dbGaP Accession phs000424.v8.p2) and experimental data [49]. Taken together, for these three pairs of genes (*DUOXA2*/*DUOX2, REG1A/REG1B,* and MUC*1*/*MUC4*), there is biologically relevant evidence in the context of IBD, and we here speculate that they also play a role in the non-inflamed ileum of UC patients, indicating the need for further studies.

An important remaining question is if the observed molecular alterations are specific to the location (ileum versus colon) or the disease (UC versus CD). While we unfortunately did not have colonic tissue available from the included patients, the identified dysregulated genes largely overlapped with those described for colonic tissue in UC [50,51,52,53], or ileum in CD [15,52,54] (Supplementary Table S6). Of the top 20 dysregulated genes in active extensive colitis, 14 showed similar findings in inflamed ileal tissue of CD [15,52,54], as well as in colonic tissue from patients with UC (13 genes) [50,51,52,53]. In addition, previous studies have reported that *MUC1*, *DUOX2*, and *DMBT1* are altered in the unaffected ileum of CD patients [55,56]. Genes dysregulated in the terminal ileum of inactive extensive colitis were also previously found to be differentially expressed in colonic tissue from patients with UC (20 of 20 genes) [50,51,52,53], and to a lesser extent in inflamed ileal tissue of CD (11 of 20 genes) [15,52,54] (Supplementary Table S6). Taken together, this suggests that some of the molecular alterations we observed in the normal ileum of active UC patients may be a shared feature of IBD, independent of the phenotype or tissue location, and thus do not represent an explanation for the spatial restriction of the inflammation in UC. It should also be noted that these molecular alterations might be associated with general intestinal inflammation itself, and not necessarily specific to UC or IBD. Additional research including the comparison with, for example, infectious colitis is needed to explore the role of these alterations in the specific context of IBD. Nevertheless, given the aberrant molecular properties of the non-inflamed ileum in UC, it is clear there is a cross-talk between the colon and ileum in UC, although we cannot currently infer a causal and/or temporal relationship between these changes and colonic inflammation. 

Even in the absence of large gene expression differences, gene co-expression network analysis can identify modules of co-expressed genes [57]. In inactive UC, we found that the correlated modules seemed to converge into intestinal barrier maintenance and mitochondrial function gene expression, and dampened immune functions. Together with multiple lines of evidence of alterations in mitochondrial function in patients with UC [51,58,59,60], this finding suggests a potential role for mitochondrial function in maintaining intestinal homeostasis. Remarkably, in general, mitochondrial function is described as decreasing in the colon of UC patients, while we found an increased function in the non-inflamed ileum of inactive UC patients. In active UC, the identified modules converged into increased immune functions. These associations can either precede (and possibly promote) colonic inflammation, be a consequence of colonic inflammation (i.e., a secondary phenomenon), or both. In conclusion, this transcriptomic analysis has identified significant molecular alterations in the non-inflamed ileum in UC. The observed changes are dependent on UC disease activity and extent (as illustrated in Figure 3), suggesting a cross-talk between the ileum and colon. With the current study, however, we cannot infer the causal and/or temporal relationship. Future longitudinal prospective studies with paired ileal and colonic samples including non-IBD controls with and without gut inflammation are therefore needed. 

## Figures and Tables

**Table 1:** Baseline characteristics.

	Active UC (*n* = 7)	Inactive UC (*n* = 29)	Control (*n* = 15)
**Male (%)**	5 (71.4)	17 (58.6)	6 (40.0)
**Median age at endoscopy (years, IQR)**	52 (36–68)	52 (33–60)	55 (46–60)
**Median disease duration (years, IQR)**	12 (6–13)	10 (8–14)	
**Maximal disease extent (%)**			
Proctitis	0	4 (13.8)	
Left-sided colitis	2 (28.6)	11 (37.9)	
Extensive colitis	5 (71.4)	14 (48.3)	
**Disease extent at endoscopy (%)**			
Left-sided colitis	4 (57.1)	N/A	
Extensive colitis	3 (42.9)	N/A	
**Concomitant medication (%)**			
Corticosteroids	1 (14.3)	0	
Immunomodulators	0	5 (17.2)	
Biologics	1 (14.3)	13 (44.8)	

UC, ulcerative colitis; IQR, interquartile range; N/A, not applicable.

**Table 2:** Significantly differentially expressed genes in the terminal ileum of UC patients.

	UC (*n* = 36) vs Control (*n* = 15)		Active UC (*n* = 7) vs Control (*n* = 15)		Inactive UC (*n* = 29) vs Control (*n* = 15)	
Gene	log_2_FC	*p*_corrected_	log_2_FC	*p*_corrected_	log_2_FC	*p*_corrected_
*DUOXA2*	2.92	0.06	**4.86**	**5.58 × 10^−3^**	2.38	0.23
*REG1B*	**3.13**	**4.85 × 10^−3^**	**4.15**	**0.02**	**2.75**	**0.03**
*PDZK1IP1*	1.12	0.08	**1.87**	**0.02**	0.94	0.23
*DUOX2*	2.39	0.09	**4.02**	**0.02**	1.39	0.63
*CASR*	−0.99	0.15	**−1.87**	**0.02**	−0.85	0.30
*CBR3*	0.70	0.13	**1.15**	**0.03**	0.58	0.31
*DMBT1*	1.48	0.11	**2.40**	**0.03**	1.14	0.34
*MUC1*	1.10	0.12	**1.84**	**0.03**	0.89	0.32
*MTRNR2L1*	0.38	1.00	**−5.94**	**0.03**	0.84	0.95
*GPR110*	1.96	0.18	**3.37**	**0.04**	1.59	0.41
*REG1A*	1.45	0.15	**2.14**	**0.04**	1.22	0.23
*NOS2*	1.37	0.23	**2.54**	**0.04**	0.99	0.61
*CFB*	0.81	0.27	**1.52**	**0.04**	0.59	0.61
*GPR37L1*	0.95	0.30	**1.77**	**0.04**	0.70	0.63
*SPINK1*	0.61	0.40	**1.30**	**0.04**	0.39	0.79
*TSPO2*	1.13	0.18	**1.82**	**0.04**	0.88	0.44
*CLDN5*	0.92	0.07	**1.31**	**0.05**	0.83	0.21
*SSTR2*	−0.83	0.12	**−1.36**	**0.05**	−0.74	0.23
*CEBPD*	**1.07**	**0.02**	0.73	0.62	**1.14**	**0.02**

Log_2_ fold-change and *p*_corrected_ for the genes that were significantly dysregulated. Significantly dysregulated genes are indicated in bold. Results in ascending order of *p*-values of the comparison between the patients with active colitis and controls. UC, ulcerative colitis; vs, versus; FC, fold-change.

**Table 3:** Significantly dysregulated genes in the terminal ileum of ulcerative colitis patients with extensive colitis (as maximal disease extent).

	Active Extensive UC (*n* = 5) vs Control (*n* = 15)		Inactive Extensive UC (*n* = 14) vs Control (*n* = 15)	
Gene	log_2_FC	*p*_corrected_	log_2_FC	*p*_corrected_
Top 20 dysregulated genes in active extensive UC				
*SPINK1*	**1.73**	**2.60 × 10^−4^**	0.31	0.77
*DUOXA2*	**5.46**	**2.81 × 10^−4^**	2.79	0.07
*PDZK1IP1*	**2.31**	**2.81 × 10^−4^**	0.81	0.37
*GPR110*	**4.22**	**8.57 × 10^−4^**	2.30	0.08
*NOS2*	**3.00**	**8.57 × 10^−4^**	1.58	0.10
*GPR37L1*	**2.07**	**8.57 × 10^−4^**	1.11	0.11
*SLC37A1*	**1.02**	**8.57 × 10^−4^**	0.39	0.35
*TFEC*	**−1.20**	**8.57 × 10^−4^**	−0.35	0.58
*CCL28*	**1.92**	**1.47 × 10^−3^**	0.51	0.67
*CFB*	**1.87**	**1.85 × 10^−3^**	0.76	0.32
*DMBT1*	**2.75**	**3.10 × 10^−3^**	1.64	0.05
*MUC1*	**2.11**	**3.44 × 10^−^** **^3^**	1.20	0.08
*PLA2G16*	**1.63**	**3.44 × 10^−3^**	0.64	0.41
*FUT3*	**1.14**	**3.44 × 10^−3^**	0.49	0.30
*NFKBIZ*	**1.46**	**3.83 × 10^−3^**	0.71	0.19
*TIMD4*	**−6.70**	**4.01 × 10^−3^**	−0.58	0.92
*DUOX2*	**4.58**	**4.49 × 10^−3^**	1.44	0.64
REG1A *	**2.44**	**4.49 × 10^−3^**	**1.65**	**0.03**
*TSPO2*	**2.13**	**4.49 × 10^−3^**	1.18	0.13
*ATP10B*	**1.49**	**4.49 × 10^−3^**	0.92	0.06
Dysregulated 20 genes in inactive extensive UC (*REG1A* is listed in the top 20 above)				
*MESDC1*	0.45	0.37	**1.08**	**2.89 × 10^−5^**
*CEBPD*	0.64	0.40	**1.46**	**6.22 × 10^−4^**
*CARD14*	1.55	0.18	**2.11**	**9.90 × 10^−3^**
REG1B *	**4.25**	**5.31 × 10^−3^**	**3.26**	**9.90 × 10^−3^**
*C2CD4B*	1.05	0.40	**2.06**	**9.90 × 10^−3^**
*NCOA7*	0.85	0.25	**1.30**	**9.90 × 10^−3^**
*CD55*	0.38	0.64	**1.23**	**0.02**
*C2CD4A*	1.72	0.18	**2.19**	**0.02**
*FAM83A*	1.63	0.23	**2.23**	**0.02**
*HES4*	0.83	0.47	**1.71**	**0.02**
GRAMD2 *	**1.97**	**0.02**	**1.53**	**0.02**
*HIC1*	0.20	0.83	**1.11**	**0.02**
*CXCL2*	2.17	0.07	**2.08**	**0.03**
*SOCS1*	0.52	0.67	**1.66**	**0.03**
MUC4 *	**2.13**	**0.02**	**1.65**	**0.03**
*TIFA*	0.81	0.44	**1.50**	**0.04**
*C4BPB*	1.03	0.13	**1.09**	**0.05**
CASP10 *	**1.35**	**0.03**	**1.03**	**0.05**
*EVA1B*	0.24	0.82	**1.17**	**0.05**

Log_2_ fold-change and *p*_corrected_ are given. Significantly dysregulated genes are indicated in bold. * Overlapping genes between active extensive UC vs control and inactive extensive UC vs control. FC, fold-change; UC, ulcerative colitis; vs, versus.

**Table 4:** Significantly correlated co-expression modules in active ulcerative colitis.

	Saddlebrown (115 genes)	Steelblue (113 genes)	Brown (781 genes)
Active UC vs CO	***r* = 0.47**	***r* = 0.49**	***r* = 0.44**
	***p* = 0.03**	***p* = 0.02**	***p* = 0.04**
	***p*_corrected_ = 0.22**	***p*_corrected_ = 0.19**	***p*_corrected_ = 0.28**
Extensive vs CO	***r* = 0.80**	***r* = 0.77**	***r* = 0.67**
	***p* = 2.78 × 10^−05^**	***p* = 6.32 × 10^−05^**	***P* = 1.17 × 10^−3^**
	***p*_corrected_ = 7.35 × 10^−3^**	***p*_corrected_ = 8.37 × 10^−3^**	***p*_corrected_ = 0.05**
Lt-sided vs CO	*r * = −0.42	*r * = −0.15	*r * = −0.34
	*p * = 0.09	*p * = 0.58	*p * = 0.18
	*p*_corrected _ = 0.37	*p*_corrected _ = 0.73	*p*_corrected _ = 0.52
Top enriched pathway in Reactome	Cytokine Signalling in Immune system	Metabolism of proteins	ER-Phagosome pathway
Top enriched pathway in IPA	Cell Death and Survival	Protein Ubiquitination Pathway	Phagosome Maturation
Top enriched GO biological process	Defence response to virus	Protein deubiquitination	Regulation of biological quality
Upstream Regulator *	IFNγ, IFNα, STAT1	EGF, EGFR, PDCD6	NFE2L2, TCR, HSPA5
Overlapping dysregulated genes	18/84 genes	-	11/84 genes

Correlation strengths r of each module were calculated for each ulcerative colitis subgroup with uncorrected/corrected *p*-values. The filled colours represent the strength of the association. A positive correlation (marked as red) means an upregulation in disease as compared to controls, while a negative correlation (marked as blue) refers to a downregulation in disease. The most highly scoring canonical pathways (according to *p*-value) according to Reactome, IPA, and GO enrichment analysis are given. * Top 3 ranked upstream regulators by IPA. UC, ulcerative colitis; CO, controls; Lt-sided, left-sided colitis as maximal disease extent; vs, versus.

## Supplementary Materials

The following are available online at https://www.mdpi.com/2073-4409/9/8/1793/s1, Figure S1: Principal component analysis plot, Figure S2. Gene expression analysis, Figure S3: Weighted gene co-expression network analysis, Table S1: Selected genes for the qRT-PCR analysis, Table S2: Significantly differentially expressed genes, Table S3: The predicted upstream regulators of differentially expressed genes, Table S4: The highly ranked pathways and gene ontology biological process, Table S5: Significantly correlated co-expression modules in inactive ulcerative colitis, Table S6: Evidence from previous studies for identified dysregulated genes in Table 3.
