# Supplementary material for: Molecular Changes in the Non-Inflamed Terminal Ileum of Patients with Ulcerative Colitis
Source: Cells. 2020 Jul 28;9(8):1793. doi: 10.3390/cells9081793 (PMC7464680; doi:10.3390/cells9081793)
Supplement: Supplementary file 1 [file cells-09-01793-s001.zip › cells-616210_cells-798328_cells-859436_supp.docx]

# Supplementary Figures and Tables

**
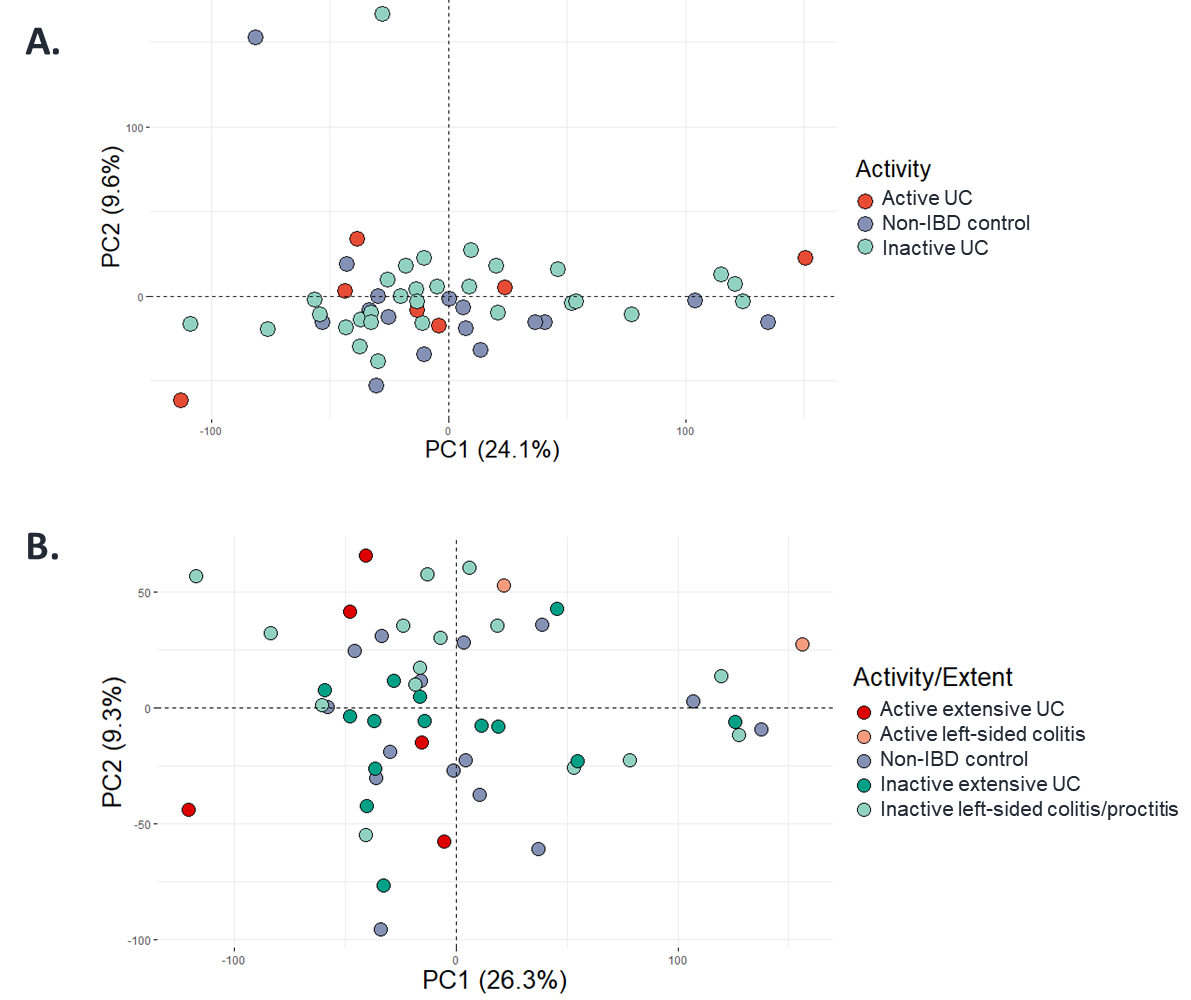
**

**Supplementary Figure 1. Principal component analysis plot.** (A) Two outlier samples (one ulcerative colitis patient and one non-IBD control) from principal component analysis were excluded. After exclusion (B), 36 ulcerative colitis patients and 15 non-IBD controls were included for further analysis.

**
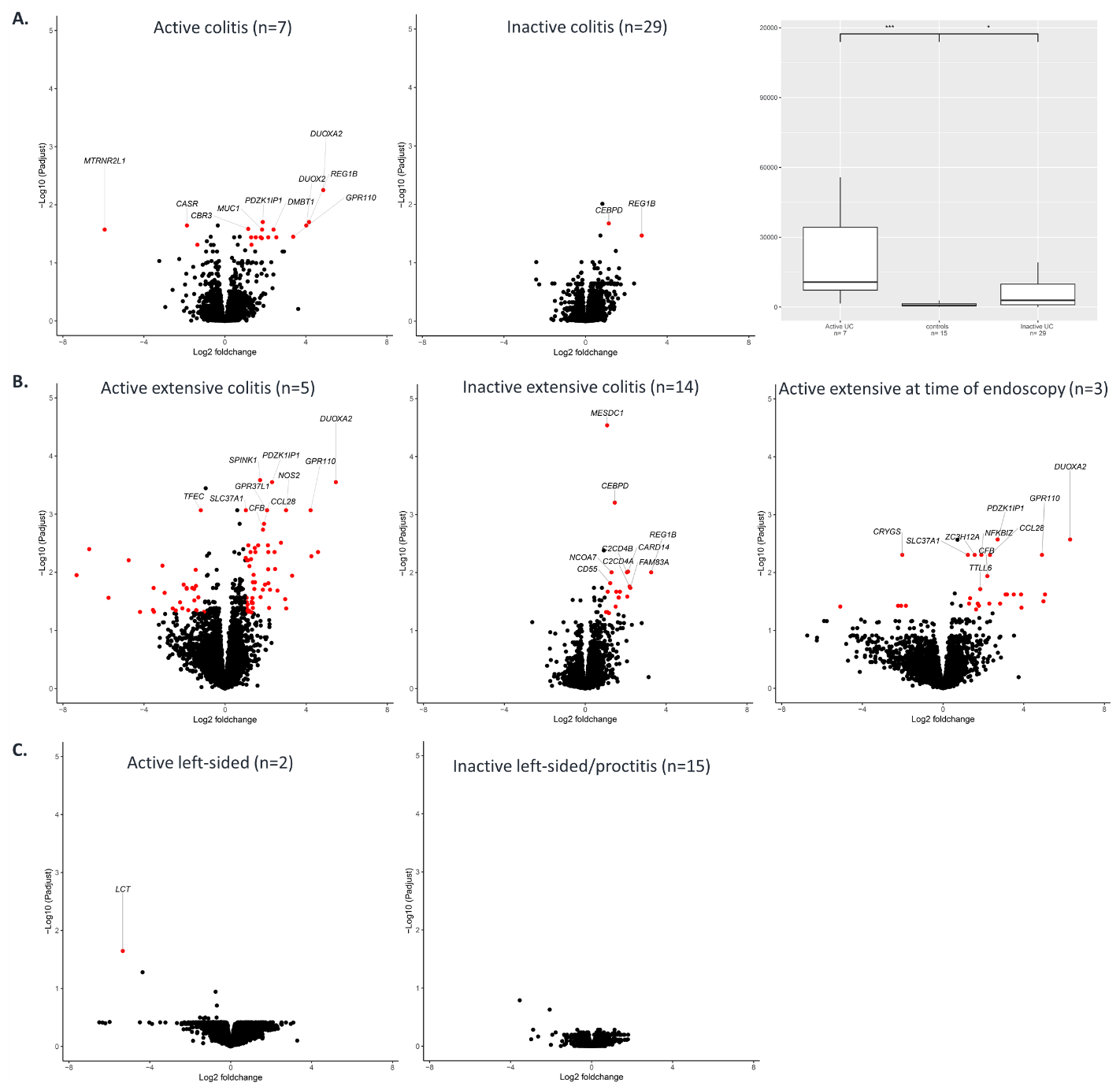
**

**Supplementary Figure 2. Gene expression analysis.** (A) Volcano plot of differentially expressed gene in ulcerative colitis with active disease versus control (left), and inactive colitis versus control (middle). Relative mRNA expression of *REG1B* in non-inflamed terminal ileum biopsies. *P-value <0.05, *** P-value <0.001. (right) (B) Volcano plots of differentially expressed gene in active extensive colitis versus control (left), inactive extensive colitis versus control (middle), and active UC with extensive extent at time of endoscopy versus control (right). (C) Volcano plot of differentially expressed gene in active left-sided colitis versus control (right) and inactive UC with left-sided colitis/proctitis versus control (middle). The red dots in the volcano plots represent significantly dysregulated genes (P_corrected_<0.05 and log_2_ fold-change≥1).

**
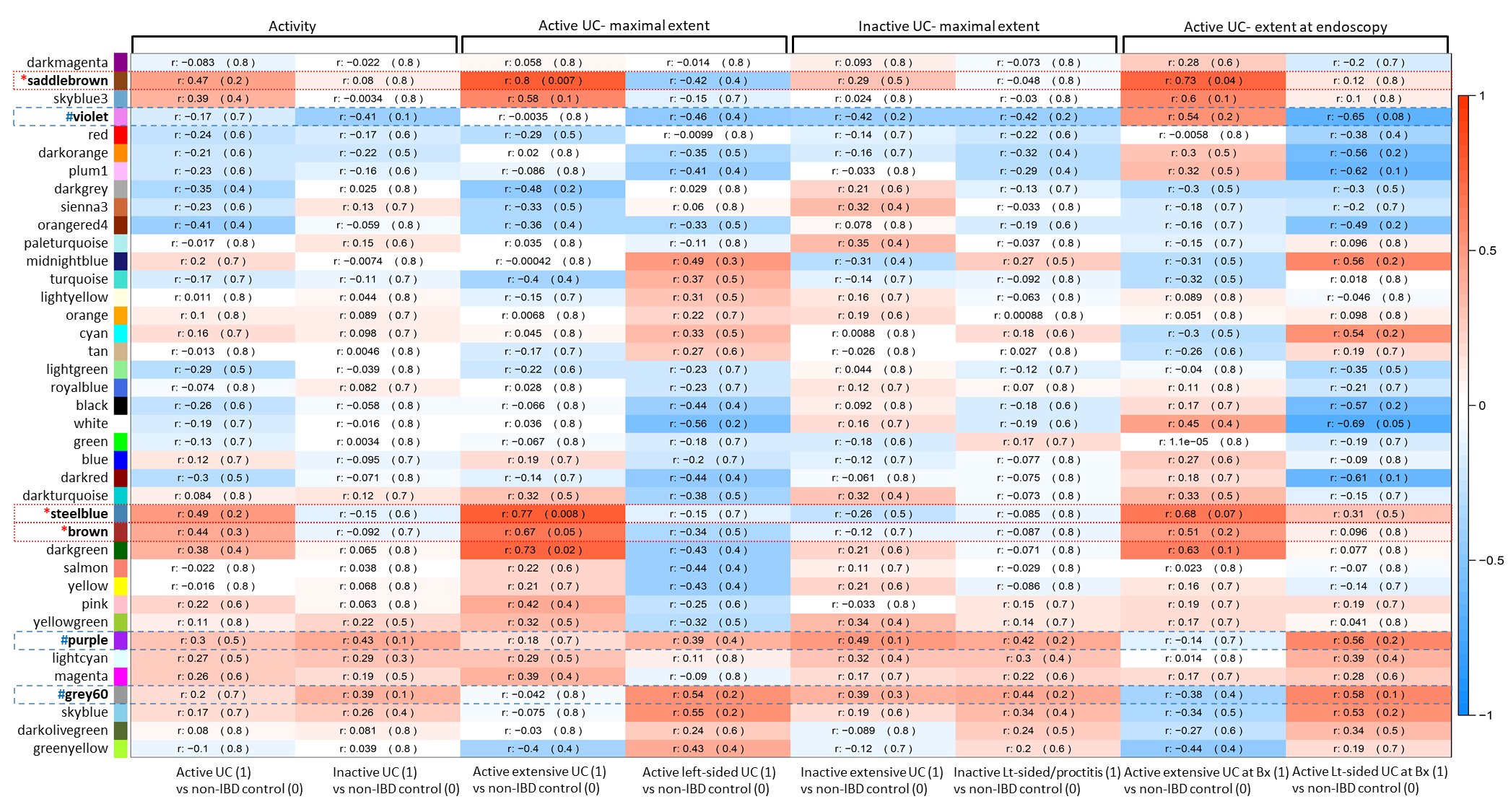
**

**Supplementary Figure 3. Weighted gene co-expression network analysis.** Co-expression modules were constructed by Weighted Gene Correlation Network Analysis (WGCNA) and correlated to disease activity, maximal disease extent during follow-up, and extent at time of endoscopy (Bx, biopsy). The heatmap represents the different modules detected (Y‐axis) and their correlation with the different traits of interest (X‐axis). Correlation strengths r of each module were calculated for each ulcerative colitis (UC) subgroup with uncorrected P-values in brackets (heatmap color represents the strength of the association: red positive and blue negative correlation in UC patients compared with controls). *Active UC-correlated modules, ^#^Inactive UC-correlated modules.

**Supplementary table 1.** Selected genes for the qRT-PCR analysis.

| Gene | TaqMan Assay ID | Amplicon Length |
| --- | --- | --- |
| *DUOXA2* | Hs01595311_g1 | 125 |
| *DUOX2* | Hs00204187_m1 | 74 |
| *REG1B* | Hs01888695_s1 | 146 |
| *REG1A* | Hs00984887_g1 | 101 |
| *MUC4* | Hs00366414_m1 | 55 |
| *GRAMD2* | Hs01584661_g1 | 94 |
| *CASP10* | Hs01017899_m1 | 74 |
| *ACTB* | Hs01060665_g1 | 63 |
| *GAPDH* | Hs99999905_m1 | 122 |

**Supplementary table 2.** Significantly differentially expressed genes.

*Data provided in separate excel file*

**Supplementary table 3.** The predicted upstream regulators of differentially expressed genes.

*Data provided in separate excel file*

**Supplementary table 4.** The highly ranked pathways and gene ontology biological process.

*Data provided in separate excel file*

**Supplementary Table 5.** Significantly correlated co-expression modules in inactive ulcerative colitis.

|  | **violet (101 genes)** | **purple (398 genes)** | **grey60 (213 genes)** |
| --- | --- | --- | --- |
| Inactive UC vs CO | **r=-0.41** | **r=0.43** | **r=0.39** |
|  | **P=6.18x10^-03^** | **P=3.88x10^-03^** | **P=9.67x10^-03^** |
|  | **P_corrected_=0.13** | **P_corrected_=0.11** | **P_corrected_=0.14** |
| Extensive vs CO | **r=-0.42** | **r=0.49** | **r=0.39** |
|  | **P=0.02** | **P=7.22x10^-03^** | **P=0.04** |
|  | **P_corrected_=0.19** | **P_corrected_=0.13** | **P_corrected_=0.26** |
| Lt/proctitis vs CO | **r=-0.42** | **r=0.42** | **r=0.44** |
|  | **P=0.02** | **P=0.02** | **P=0.02** |
|  | **P_corrected_=0.19** | **P_corrected_=0.19** | **P_corrected_=0.19** |
| Top enriched pathway in Reactome | Signal regulatory protein family interactions | Mitochondrial translation | ­- |
| Top enriched pathway in IPA | Lymphotoxin β Receptor Signaling | Planar cell polarity (PCP) pathway | Regulation of Actin-based Motility by Rho |
| Top enriched GO biological process | ­- | Mitochondrial translational termination | ­- |
| Upstream Regulator* | RAB1B, TACC3, ETV4 | TOX, MAPK9, AFAP1-AS1 | RNF20, CDK4/6,CAF-1 |
| Overlapping dysregulated genes | ­- | 6/20 genes | ­- |

Correlation strengths r of each module were calculated for each ulcerative colitis subgroup with uncorrected/corrected P-values. The filled colours represent the strength of the association. A positive correlation (marked as red) means an upregulation in disease as compared to controls, while a negative correlation (marked as blue) refers to a downregulation in disease. The highly scoring canonical pathways (according to P value) according to Reactome, IPA, and GO enrichment analysis, respectively. ^*^Top 3 ranked upstream regulators by IPA. UC, ulcerative colitis; CO, controls; Lt/proctitis, left-sided colitis/proctitis as maximal disease extent; vs, versus.

**Supplementary table 6.** Evidence from previous studies for identified dysregulated genes in Table 3.

| **Disease** | **Ulcerative colitis** | | | | **Crohn's disease** | | | |
| --- | --- | --- | --- | --- | --- | --- | --- | --- |
| Location | Colon [1] | Colon [2] | Colon [3]^$^ | Colon [4]^#^ | Ileum [5] | Ileum [6] | | Ileum [3]^$^ |
| Studied group | Newly diagnosed | Newly diagnosed | Active UC | Active UC | Newly diagnosed | Newly diagnosed | Newly diagnosed | Active CD |
| Top 20 dysregulated genes in active extensive UC | | | | | | | | |
| *SPINK1* | 1.60 |  | 1.20 |  |  |  |  |  |
| *DUOXA2* | 7.21 | 7.71 | 6.13 | 4.32 | 2.91 | 5.45 | 5.65 | 5.56 |
| *PDZK1IP1* | 2.18 | 1.93 | 3.29 | 1.92 | 1.46 | 1.80 | 1.59 | 3.36 |
| *GPR110* |  |  |  |  | 1.67 |  |  |  |
| *NOS2* | 4.03 | 3.61 | 3.44 | 3.35 | 2.19 | 1.73 | 2.69 | 2.77 |
| *GPR37L1* |  |  |  |  |  |  |  |  |
| *SLC37A1* |  |  |  |  |  |  |  |  |
| *TFEC* |  | 2.04 | 1.81 |  |  |  |  | -1.99 |
| *CCL28* |  |  |  |  | 1.48 | 1.21 | 1.58 | 1.84 |
| *CFB* | 3.21 | 2.86 | 2.88 | 2.33 | 1.09 | 0.87 | 1.08 |  |
| *DMBT1* | 3.49 | 4.40 | 3.14 | 2.62 | 1.42 | 1.35 | 1.70 | 2.09 |
| *MUC1* | 1.39 | 0.98 | 1.48 | 1.20 | 3.11 | 3.34 | 3.27 | 4.11 |
| *PLA2G16* |  | 1.07 | 1.19 |  |  |  |  |  |
| *FUT3* |  |  |  |  |  |  |  | 1.10 |
| *NFKBIZ* | 1.44 | 1.87 | 1.59 | 1.40 | 1.31 | 1.07 | 1.53 | 1.78 |
| *TIMD4* |  |  |  |  |  |  |  |  |
| *DUOX2* | 5.73 | 5.59 | 5.00 | 4.34 | 4.33 | 3.84 | 4.59 | 6.48 |
| *REG1A** | 5.05 | 9.14 | 7.88 | 4.63 |  | 1.07 | 1.31 |  |
| *TSPO2* |  | 2.73 | 1.83 |  |  |  |  |  |
| *ATP10B* |  |  |  |  |  |  |  | 1.24 |
| Dysregulated 20 genes in inactive extensive UC (*REG1A* is listed in the top 20 above) | | | | | | | | |
| *MESDC1* |  |  | 1.09 |  |  |  |  |  |
| *CEBPD* |  | 0.96 | 1.42 |  |  | 0.87 | 1.08 |  |
| *CARD14* |  | 1.75 |  |  |  |  |  |  |
| *REG1B** | 3.73 | 7.31 | 5.60 | 4.08 | 2.30 | 1.17 | 2.05 |  |
| *C2CD4B* | 2.79 | 2.25 | 1.63 |  |  |  |  |  |
| *NCOA7* | 1.37 | 1.55 | 1.47 | 1.21 |  |  |  |  |
| *CD55* | 1.89 | 2.62 | 3.44 | 2.30 | 1.26 | 1.18 | 1.35 | 1.98 |
| *C2CD4A* | 3.72 | 3.70 | 5.03 |  |  | 2.05 | 2.15 | 3.57 |
| *FAM83A* | 4.11 | 4.74 | 2.19 |  |  |  |  | 1.64 |
| *HES4* |  | 1.44 |  |  |  |  |  |  |
| *GRAMD2** |  |  | 2.46 |  |  |  |  |  |
| *HIC1* |  | 1.35 |  |  |  |  |  |  |
| *CXCL2* | 3.07 | 3.76 | 4.29 | 2.38 |  | 2.40 | 2.69 | 2.82 |
| *SOCS1* | 2.77 | 2.38 | 2.06 | 1.15 |  | 0.72 | 1.34 |  |
| *MUC4** | 1.23 | 1.59 | 1.64 |  | 2.39 | 2.42 | 2.46 | 3.71 |
| *TIFA* |  | 1.07 | 1.65 |  |  | 0.64 | 1.02 |  |
| *C4BPB* | 3.58 | 4.15 | 4.15 | 2.14 |  | 1.86 | 1.88 | 2.38 |
| *CASP10** | 1.01 | 1.05 |  |  |  |  |  |  |
| *EVA1B* |  | 1.35 |  |  |  |  |  |  |

Log_2_ fold-changes for dysregulated genes based on same threshold as our study (P_corrected_<0.05 and log_2_ fold-change≥1) are given. *Overlapping genes between active extensive UC vs control and inactive extensive UC vs control. ^$,#^Differences calculated using GEO2R (GSE16879^$^ and GSE59071^#^, respectively). UC, ulcerative colitis; CD, Crohn's disease.

1. Taman, H.; Fenton, C.G.; Hensel, I.V.; Anderssen, E.; Florholmen, J.; Paulssen, R.H. Transcriptomic Landscape of Treatment-Naive Ulcerative Colitis. *J Crohns Colitis* **2018**, *12*, 327-336.

2. Haberman, Y.; Karns, R.; Dexheimer, P.J.; Schirmer, M.; Somekh, J.; Jurickova, I.; Braun, T.; Novak, E.; Bauman, L.; Collins, M.H., et al. Ulcerative colitis mucosal transcriptomes reveal mitochondriopathy and personalized mechanisms underlying disease severity and treatment response. *Nat Commun* **2019**, *10*, 38.

3. Arijs, I.; De Hertogh, G.; Lemaire, K.; Quintens, R.; Van Lommel, L.; Van Steen, K.; Leemans, P.; Cleynen, I.; Van Assche, G.; Vermeire, S., et al. Mucosal gene expression of antimicrobial peptides in inflammatory bowel disease before and after first infliximab treatment. *PLoS One* **2009**, *4*, e7984.

4. Vanhove, W.; Peeters, P.M.; Staelens, D.; Schraenen, A.; Van der Goten, J.; Cleynen, I.; De Schepper, S.; Van Lommel, L.; Reynaert, N.L.; Schuit, F., et al. Strong Upregulation of AIM2 and IFI16 Inflammasomes in the Mucosa of Patients with Active Inflammatory Bowel Disease. *Inflamm Bowel Dis* **2015**, *21*, 2673-2682.

5. Verstockt, S.; De Hertogh, G.; Van der Goten, J.; Verstockt, B.; Vancamelbeke, M.; Machiels, K.; Van Lommel, L.; Schuit, F.; Van Assche, G.; Rutgeerts, P., et al. Gene and Mirna Regulatory Networks During Different Stages of Crohn's Disease. *J Crohns Colitis* **2019**.

6. Haberman, Y.; Tickle, T.L.; Dexheimer, P.J.; Kim, M.O.; Tang, D.; Karns, R.; Baldassano, R.N.; Noe, J.D.; Rosh, J.; Markowitz, J., et al. Pediatric Crohn disease patients exhibit specific ileal transcriptome and microbiome signature. *J Clin Invest* **2014**, *124*, 3617-3633.
